# Development of data representation standards by the human proteome organization proteomics standards initiative

**DOI:** 10.1093/jamia/ocv001

**Published:** 2015-02-28

**Authors:** Eric W Deutsch, Juan Pablo Albar, Pierre-Alain Binz, Martin Eisenacher, Andrew R Jones, Gerhard Mayer, Gilbert S Omenn, Sandra Orchard, Juan Antonio Vizcaíno, Henning Hermjakob

**Affiliations:** 1 ^1^Institute for Systems Biology, Seattle, USA; 2 ^2^Proteomics Facility, Centro Nacional de Biotecnología - CSIC, Madrid, Spain; 3 ^3^ProteoRed Consortium, Spanish National Institute of Proteomics, Madrid, Spain; 4 ^4^CHUV Centre Hospitalier Universitaire Vaudois, Lausanne, Switzerland; 5 ^5^Medizinisches Proteom Center (MPC), Ruhr-Universität Bochum, Bochum, Germany; 6 ^6^Institute of Integrative Biology, University of Liverpool, Liverpool, UK; 7 ^7^Department of Computational Medicine & Bioinformatics, University of Michigan, Ann Arbor, USA; 8 ^8^European Molecular Biology Laboratory, European Bioinformatics Institute (EMBL-EBI), Wellcome Trust Genome Campus, Hinxton, Cambridge, UK; 9 ^†^Died July 18, 2014

**Keywords:** standards, data standards, data formats, guidelines, proteomics, standards organization, HUPO, proteomics standards initiative

## Abstract

**Objective** To describe the goals of the Proteomics Standards Initiative (PSI) of the Human Proteome Organization, the methods that the PSI has employed to create data standards, the resulting output of the PSI, lessons learned from the PSI’s evolution, and future directions and synergies for the group.

**Materials and Methods** The PSI has 5 categories of deliverables that have guided the group. These are minimum information guidelines, data formats, controlled vocabularies, resources and software tools, and dissemination activities. These deliverables are produced via the leadership and working group organization of the initiative, driven by frequent workshops and ongoing communication within the working groups. Official standards are subjected to a rigorous document process that includes several levels of peer review prior to release.

**Results** We have produced and published minimum information guidelines describing what information should be provided when making data public, either via public repositories or other means. The PSI has produced a series of standard formats covering mass spectrometer input, mass spectrometer output, results of informatics analysis (both qualitative and quantitative analyses), reports of molecular interaction data, and gel electrophoresis analyses. We have produced controlled vocabularies that ensure that concepts are uniformly annotated in the formats and engaged in extensive software development and dissemination efforts so that the standards can efficiently be used by the community.

**Conclusion** In its first dozen years of operation, the PSI has produced many standards that have accelerated the field of proteomics by facilitating data exchange and deposition to data repositories. We look to the future to continue developing standards for new proteomics technologies and workflows and mechanisms for integration with other omics data types. Our products facilitate the translation of genomics and proteomics findings to clinical and biological phenotypes. The PSI website can be accessed at http://www.psidev.info.

## INTRODUCTION

Mass spectrometry (MS) proteomic identification of the protein content of clinical samples is of increasing importance in laboratory medicine. MS-based proteomic measurements have been used in a wide range of biological and biomedical studies, including the identification and monitoring of markers of disease onset and progression, toxicology studies, and monitoring of drug responsiveness. MS proteomics encompasses a broad range of separation and identification techniques and is performed on many different instrumentation and software platforms. Research in this field therefore needs to be supported by a robust data analysis pipeline to process the many data types generated at various stages of the analysis process.

It has been broadly recognized that common data standards are a crucial element of advancing a research field.^1–5^ The many benefits include the enhanced interoperability of software tools, greater usability of tools across different instrument vendors and computer operating systems, and the increased ease of sharing, reusing, and depositing data in public repositories. Minimum information (MI) guidelines describe what data elements and metadata are to be provided when making data public, while data formats are the data models for encoding the information that is to be shared. Formats may be broadly categorized as *ad hoc* formats, which are quickly implemented to meet immediate needs of one group or developer, *de facto* standards, which have never been through a standardization process but have nonetheless become ubiquitous, and true standards, which are created through a formal standardization process with the input of many. While standards and common formats are a benefit to a field, there can exist too much of a good thing, in the form of excess and uncoordinated standards or formats, which can cause confusion in the community and extra work for software developers, who must attempt to support extra formats.

In an attempt to coordinate data standards for the burgeoning field of proteomics, the Human Proteome Organization (HUPO) formed the Proteomics Standards Initiative (PSI) in 2002. The PSI immediately began work to develop standard formats to encode the output of mass spectrometers and the output of the subsequent informatics analysis. It also began to develop a minimum information guideline to describe what information should be provided when making MS proteomics data public.

MS proteomics has become the most widely used technique for detecting and quantifying the abundance of multiple proteins in complex samples. The basic workflow is to extract proteins from a (potentially) complex biological sample, digest the proteins into peptides with an enzyme (usually trypsin), fractionate the sample to reduce complexity, and separate the peptides in each fraction via liquid chromatography so that only a few peptides are ionized and introduced into the mass spectrometer at a time. The instrument acquires a mass spectrum to determine the mass-to-charge ratio (*m/z*) of all ions entering the instrument at each time point. In many workflows, 1 or more of these ions are isolated by the instrument, fragmented in a collision cell, and then analyzed to yield a fragmentation spectrum of just the isolated species. The resulting mass spectra (or the chromatograms in some workflows) must then undergo extensive informatics analysis via sophisticated algorithms to yield protein identifications and quantitative abundance measures.^6^ Many variations on this theme exist, limited only by the continually advancing technology of mass spectrometry (MS) instruments and the creativity of scientists and software developers to analyze the complex data into usable results.

Since there are hundreds of software tools,^7^^,^^8^ both commercial and free, that are routinely used to process the data being generated by instruments of many different types, offered by half a dozen major manufacturers, the field of MS proteomics must have well-defined standards if these tools are to interoperate and the field is to advance rapidly. The PSI has taken a very active role in defining standards for MS proteomics and molecular interaction data over the past dozen years, serving not only to define the standards used by many of the software tools in the field but also to bring together the software developers so that they exchange ideas and consider interoperability issues more readily. Although the most active members of the PSI have changed considerably over the past dozen years, the group has successfully developed many influential standards that have enabled the interoperability of many software tools and resources.

In the field of molecular interactions, the work of the PSI has mainly centered on standardizing the download format of the many protein interaction databases that currently exist such as IntAct,^9^ Molecular INTeraction (MINT),^9^ and Database of Interacting Proteins (DIP).^10^ Standardized data have allowed the user to merge data from multiple resources relatively easily and enabled the development of visualization and analysis tools that operate over datasets derived from many disparate sources.^11^^,^^12^ For example, the user may assemble an integrated human interactome and perform sophisticated network analyses of clinical proteomic data using packages such as the Cytoscape data integration, analysis, and visualization package.^13^

We describe the methods that the PSI has employed to create its standards, summarize the results of the PSI, discuss the lessons learned from the PSI’s evolution, and describe future directions and synergies for the group. This article is distinguished from other PSI publications in that it gives a complete perspective on the methodologies of the PSI, its past successes and future challenges, whereas previous publications have described individual standards, resources, or workshops.

## METHODS

### Organizational Structure

The PSI has 5 categories of deliverables that have guided the group. These are minimum information guidelines, data formats, controlled vocabularies (CVs), resources and software tools, and dissemination activities. The minimum information guidelines are developed in broad discussions with many researchers in the field. The data formats are developed primarily by bioinformaticians to support tools but are guided significantly by the minimum information guidelines to ensure that the formats can encode all the specified information and more. The CVs are developed specifically to support the formats so that a wide variety of concepts can be encoded in flexible formats using the same vocabulary. The resources and tools are created not just by those actively participating in the PSI but also by other developers willing to support the community-developed standards. Finally, the members of the PSI foster many activities to inform the community on the products of the PSI and how they can make data analysis and sharing easier for everyone. Each of these categories will be discussed in more detail in the Results section below. The PSI has a leadership team, the steering committee, that guides the activities of the group. There is a single chair, assisted by 2 co-chairs. The steering committee is then completed with 1 or 2 members each that fulfill the roles of editor, secretary, and primary domain coordinators for minimum reporting requirements, CVs, and website content. The PSI is further organized into working groups that produce the deliverables in several different categories of proteomics data. Current workings groups (WGs) are the MS Standards WG, Proteomics Informatics WG, Molecular Interactions WG, Protein Modifications WG, and Protein Separation WG. Each WG has members who fill the same roles as listed above for the steering committee (chair, co-chair, editor, etc). For an updated list of the people fulfilling the different roles, see http://www.psidev.info/roles.

Most of the participants in the PSI are academic researchers and bioinformatics software developers. However, a key component for the PSI has been the involvement of instrument vendors, commercial software vendors, and journal editors. The instrument vendors have an interest in ensuring that data from their machines are compatible with a wide variety of software. Commercial software vendors also seek the broadest application of their software, and open formats reduce the number of formats they must support in order to make their software attractive to most users. Finally, the journal editors have an interest in making the data that support the studies they publish as openly accessible as possible, and this is greatly facilitated by minimum information guidelines, open data format standards, and the many tools that support them. This helps reviewers to spot-check the data supporting claims made in the manuscript and fosters reuse of published data, which yields greater citations to the original publications. Senior editors are typically present at PSI workshops, and the PSI has hosted several smaller workshops specifically to gather input on the PSI guidelines. There is no formal membership process for participants, and therefore the total size of the PSI is uncertain. However, several hundred workers have contributed to the PSI over the past dozen years. The current count of the union of various active PSI email distribution lists is over 250 members.

The PSI website (http://www.psidev.info) provides extensive information on all of the activities of the PSI, including information about the leadership, upcoming events, and working groups. The most extensive aspect of the website is to provide information and documentation for the products of the PSI. Most of the formats and products of the PSI are published in journal articles, but only a limited amount of information can be provided in journal articles. Accompanying each article are extensive documentation and guidance for using these products in the form of full specification documents, detailed examples, online documentation, and example files and discussion lists that document the ongoing questions, answers, and discussions that took place during and after development of the standards. Maintenance of a website to keep the information fresh is always a challenge, but several members within the PSI are tasked with this effort.

### Organizational Processes

One of the main driving forces behind the successes of the PSI has been the periodic multiday workshops. In the early years of the PSI, they occurred twice per year, but as our experience and communication has evolved, these are now held once per year in the spring. These workshops are multiday events with several goals. The main goal is to get the most active members together to meet face to face and discuss the most important open issues and, most importantly, arrive at decisions. Perhaps the greatest challenge of standards development is that there is often more than one way that information could be encoded or described, with different participants promoting different approaches. In the end, the formats should allow just a single way to do it; thus, consensus must be achieved after considering the options. This is most easily done face to face. Further goals of the workshops are to bring new participants into the PSI. In an attempt to foster more local participation, the workshops are typically held in different parts of the world in different years. Ideally, some of these local participants are then motivated to begin long-term participation in the PSI. Finally, the workshops also serve to educate those not yet familiar with the work of the PSI and those struggling to implement the standards in their own software or workflows. Easy access to many of the developers of the standards and their own software tools can be very beneficial for such participants. In all, the workshops achieve several goals each year, and a conference report has been published for each of them.^14–21^

However, most of the progress on standards cannot happen in a few short days every year. Once the decisions are made, there is significant additional work that needs to be done by individuals, with the results to be validated by the group, including documentation work, software development, and generation of examples. Ongoing communication amongst the working groups is achieved via scheduled telephone conferences to review progress and action items and spur continued development. In some cases, special smaller workshops are called to bring together the main contributors to 1 specific subtopic in an effort to push forward the completion of a standard. In addition, the PSI typically hosts a special session at the HUPO World Congress every year (usually held in September or October). This session serves to promote the PSI by presenting to a larger audience the progress and plans of the PSI and fostering discussion among session participants who do not frequently participate in other PSI events.

The 2 primary factors that set the standards developed by the PSI apart from other formats in use are the many viewpoints that go into the development and the official process by which the end product is approved as a standard. The mechanism by which standards are approved by the PSI is called the Document Process. For each new standard, a formal specification document is prepared along with a minimum of 3 example files. The document and examples are submitted to the PSI Editor. The editor checks that the submission is suitable and then passes it to the steering committee for an initial review. After the steering committee review, any requested changes are made. Following this, the editor seeks peer reviewers from the community to review the standard as they would a journal article, after which the submitters address the reviewers’ comments. Finally, there is an open community review period, during which the draft is well advertised and anyone in the community is encouraged to provide feedback. At the end of this process, after all feedback has been addressed (usually after a few iterations), the specification is approved and it becomes an official standard of the PSI. This process is described in detail by Vizcaíno *et al.*^22^ In some cases, this process is performed in parallel with the review of a manuscript that is submitted to a journal. This process ensures that all products of the PSI are well reviewed and reflect the best wisdom of many in the community.

In addition to creating new standards, an important ongoing role for the PSI is to maintain its existing standards so they remain applicable for new workflows, applications, and technologies. The PSI remains committed to maintaining its standards so that they can continue to be used for their original purpose and can be extended to new techniques as appropriate. It is not uncommon for developers of new techniques to hack in changes to existing formats or invent their own formats if the currently available formats appear to be deficient for supporting the technique. However, this then hinders the adoption of the new technique, since existing software and tools cannot interoperate with the *ad hoc* formats. As such, the PSI periodically updates the formats to properly handle new techniques, and some of these updates are also published in scientific journals. In most cases, efforts are made to leave the schema unchanged and support the new information via new CV terms. When the necessary changes are too extensive for simple CV additions, then the schema version is updated and a revised specification document is produced and submitted to the document process to undergo review as described above.

## RESULTS

### Minimum Information Guidelines

The PSI has developed several minimum information guidelines in the past dozen years. They were initially modeled after the successful Minimum Information About a Microarray Experiment guidelines.^23^ Our goal has been to define the minimum amount of information needed to understand how a study was executed and reproduce it if needed. There is a strong tendency when many PSI participants collaborate on this process to identify many pieces of information that would be beneficial in some circumstances to know, and it becomes difficult to winnow down all the suggestions to a set that is truly minimal and not overly burdensome. It is important to highlight that in its minimum information guidelines, the PSI has never tried to stipulate how any experiment should be done but rather just require full disclosure of what was done. For example, there is no language that specifies how many replicates must be performed or what false discovery rate threshold to apply but rather just how many replicates were performed and what threshold was applied and the method used so that reviewers or receivers of data may then better judge if the quality is sufficient.

The Minimum Information About a Proteomics Experiment (MIAPE) guideline was first developed by the PSI in 2006.^24^ It was designed to be modular in structure so that the many possible facets of varied and complex proteomics workflows could be captured in different modules.^25^ The original paper^24^ describes the general philosophy and structure of the modules. Collectively, the modules provide guidelines for such information as experimental design, hardware specifications, instrument parameters, software versions and parameters, and analysis choices. MIAPE-CC^26^ describes the details of column chromatography. MIAPE-GE and MIAPE-GI describe the minimal reporting requirements for running gel electrophoresis and gel electrophoresis image analysis, respectively.^27^ MIAPE-MS^28^ describes the minimum information for running the mass spectrometer and generation of the raw data. The MIAPE-MSI^29^ module describes the information needed for the complex informatics workflow used to analyze the mass spectra. The most recent one is the MIAPE-Quant^30^ module, which describes all the aspects of the quantitative part of a study, from labeling chemistry to informatics analysis. Although the multiplicity of these modules adds complexity, it has the advantage that only the modules that are applicable need be selected. A few of these guidelines have been updated since the original versions were published.

The MIAPE guidelines have been implemented in the ProteoRed tools^31^ but not in most other tools in a complete manner, partly due to the complexity in capturing all these metadata. In many cases, the information is being captured in the manuscript describing a dataset but is not yet encoded in a parsable form. The PSI formats do support the encoding of the minimum information in the formats but do not yet require it. In some cases, the official validation software for a format is able to check that MIAPE guidelines have been met, eg, mzIdentML.^32^ In some cases, journals have developed their own guidelines,^2^^,^^33^ which tend to also require minimum-quality criteria in an effort to promote high-quality results and minimize the publication of false positives.

The PSI has also developed the Minimum Information about a Molecular Interactions Experiment guidelines,^34^ primarily aimed at improving the quality of experimental detail supplied by authors in manuscripts and to encourage the deposition of data into databases as part of the publication process. In practice, this has become a recognized descriptor for the amount of information supplied by many protein interaction databases. Related standards, detailing the information required to describe a protein affinity reagent (MIAPAR^35^) or drug-like molecules used in drug-target studies (Minimum Information About a Bioactive Entity^36^), were subsequently published by this group.

### Standard Formats

The 2 broad types of standard formats from the PSI are complex formats encoded in Extensible Markup Language (XML) and simplified summary formats encoded in tab-separated-value tabular formats. The XML formats aim to encode nearly complete data and metadata in a flexible fashion using a CV so that new technologies and workflows can be supported via new CV terms without a change in the XML schema. This heavy use of CV terms provides the great benefit of flexibility but comes at a cost of inflated file sizes and risk of different “dialects”—that is, different possibilities of encoding the same information. In this subsection, we will provide a brief history of the evolution of the PSI development process and an overview of the formats that the PSI has produced.

At the beginning of the PSI, it was well known that an open vendor-neutral format for storing the output of mass spectrometers was needed. Although simple text file formats for MS2 (fragmentation) spectra were numerous and common, attempts to write quantification software that needed access to the MS1 scans were severely hindered by a lack of a common open format. The PSI began development of such a format, but due to general inexperience with developing complex XML formats and many varied opinions, progress was slow. At the Institute for Systems Biology (ISB, Seattle, Washington), lack of an open format for all output of a mass spectrometer seemed so acute that researchers there developed their own format, mzXML.^37^ Since the format needed only to meet ISB’s needs, the format was simpler and was completed far more quickly than the PSI’s mzData effort. The mzData was eventually finished and released but not before mzXML had become widely used by many pieces of software.

It was then widely recognized that having 2 different XML-based formats for the same data, output of mass spectrometers, was also a hindrance. There was confusion in the community about which format to use. Developers needed to choose which format to use or expend extra effort to support both. Therefore, at the 2006-PSI spring workshop, the developers of PSI’s mzData and developers of ISB’s mzXML, along with instrument and software vendor developers, set out to create a new format that brought together all the best aspects of the 2 formats.^38^ The resulting mzML format^39^ was finished and released in 2008. It was regarded widely as a significant improvement over the 2 precursor formats. This format is now widely used and quite stable, although use of mzXML has still not been fully discontinued.

The TraML format^40^ was developed using a similar technology (a flexible XML format constrained heavily with CV elements) as mzML. However, where mzML is designed for mass spectrometer output, TraML is designed for mass spectrometer target input. For data-dependent acquisition modes, the instrument itself decides which ions to select for fragmentation. However, for several data-independent acquisition (DIA) modes such as selected reaction monitoring (SRM) and inclusion-list based workflows, the instrument is given a list of targets for which data should be acquired. TraML is able to encode these inputs for mass spectrometers, both inclusion lists and SRM transition lists. TraML can optionally also encode extensive metadata on the lists to indicate provenance and other instrument optimizations for individual elements in the list.

While the mzML format was designed to encode the mass spectra produced by all types of instruments used in the field, it was explicitly not designed to capture any downstream interpretation of the spectra. The PSI has designed a pair of XML formats to capture this downstream processing. The mzIdentML format^41^ was released in 2011 and encodes the results of software that identifies the peptide ions thought to be represented by each spectrum as well as statistical validation of those putative identifications. In addition, it can capture the protein identifications derived from those detected peptides, for which guidelines have recently been updated and improved.^42^ The mzQuantML format,^43^ released in 2013, was designed to encode the quantitative component of an experiment if it was performed. The mzQuantML format is very flexible and can encode all types of quantitation results, including those from isotopic labeling, isobaric labeling, label-free intensity-based quantitation, spectral counting, and SRM/DIA quantitation. Although these 2 components were together in a single format in early design, it was realized that the scope was too large and progress would be better with 2 separate formats.

These 2 XML-based formats can very flexibly capture the results of many diverse workflows along with rich metadata on how the results were obtained. The downside is the significant complexity in reading and writing these formats. It has become common to export the highest-level results from these formats into simple text files that can be easily ingested into tools such as MS Excel (Microsoft, Redmond, Washington) and the programming language R^44^ for further analysis. To meet this need, the PSI recently developed the mzTab format,^45^ a tab-delimited format that can encode the highest-level results from both mzIdentML and mzQuantML into a single file. There were misgivings that providing this simpler format would discourage the usage of the more complete XML formats. In the end, it seemed better to standardize what seemed like an inevitable use case rather than withhold such a format in an effort to promote mzIdentML and mzQuantML.

As described above, another major product of the PSI has been the development of formats to support the exchange of molecular interactions data. The PSI-MI XML format^12^ has evolved significantly over the past 10 years to capture rich information about interactions between molecules of all different types, including metabolites, DNA, proteins, and protein complexes. As with other PSI formats, it has become quite complex in the data it is able to encode, and a simpler tab-separated format called MITAB^35^ has been developed to support use cases where simplified metadata is sufficient. Due to user demand, this now exists in 3 versions (MITAB 2.5, 2.6, 2.7) with progressively more columns supplying additional information about the interaction being studied.^36^

The PSI has developed some additional formats to capture information about specific aspects of some proteomics workflows that cannot be captured in the above formats. The GelML format^46^ encodes information about the detection, characterization, and quantification of gel spots and bands prior to excision and analysis. The spML format captures information about molecule separation steps, primarily the separation of peptides via column chromatography. However, it should be noted that GelML and spML are not widely used, and their further development has been discontinued. The qcML format^47^ enables the consistent encoding and exchange of quality control metrics derived from periodic quality control runs on an instrument; it has not yet been approved as a PSI standard but follows the same style and conventions as the PSI standards.

Finally, the PSI Extended FASTA Format is still in development and aims to extend the ubiquitous FASTA format by imposing a specific syntax in the description field of the FASTA format. This syntax enables the consistent parsing of metadata for each protein such as species, names, accessions, known sequence variants, and known post-translational modifications, which can then be used by search engines and other downstream processing of the data.

### Controlled Vocabularies

The PSI has developed several CVs^48^ primarily to correspond to the formats. The PSI formats are generally quite flexible in the metadata that can be encoded. Rather than having metadata such as instrument models, software names, and instrument configuration modes enumerated in the XML schema, which would then require frequent schema updates, most of the PSI formats allow the flexible use of terms from a CV to be specified. This allows new concepts and instances to be flexibly added to the CV and then to documents while still ensuring that there is only a single way to refer to a specific concept. We make the distinction between an ontology and a CV such that an ontology has a very carefully constructed hierarchical structure of terms with “is a” and “part of” relationships to join them, whereas a CV focuses on the terms and definitions with less regard to their semantic relationships. The PSI CVs are managed by committees within the PSIs and generally maintained in Open Biomedical Ontology (OBO) format, which is easily machine-parsable and supports both CVs and ontologies.

The PSI-MS CV^49^ contains most of the vocabulary terms used by the mzML, mzIdentML, mzQuantML, mzTab, and TraML formats, and it is also used by other databases and resources such as the PRIDE database.^50^^,^^51^ It includes terms for all commonly used mass spectrometers, data processing software, and data scoring metrics that must be reported in the various formats. This ensures that all these concepts are written consistently and can be interpreted consistently by parsing software. The PSI-MS CV is often updated because additions to the CV are requested by the community on a regular basis. The formal process followed to update the PSI-MS CV is described in detail in Mayer *et al.*^49^ There is very little overlap between the PSI-MS CV and other CVs or ontologies, with the exception of the IUPAC list of MS terms,^52^ which is itself not accession number based in a manner that is easily parsed by software. It is rather a wiki-based list of terms and definitions, and where there is overlap, we have attempted to synchronize the terms and definitions so they are fully compatible.

The PSI-MI CV contains the many terms that are required to encode molecular interactions in the PSI-MI XML and MITAB formats. These terms pertain only to concepts and resources relevant to molecular interactions and do not overlap with PSI-MS terms. Additionally, PSI-MOD^53^ is a well-structured ontology of protein, peptide, and amino acid modifications, both natural and artefactual, and is used by both MS and MI data resources and increasingly by other protein-centric data resources. Several other minor CVs are in use by minor formats; details may be found at the PSI website.

### Databases and Tools

The development and use of such standard formats, minimum information guidelines, and CVs can have a tremendously beneficial effect on the advancement of a field. However, the adoption of these standards is directly dependent on the quality of software that implements these standards. If high-quality and easy-to-use software enables the use of the standards, the benefit of standards is realized. If there is no such software, then the standards will be largely ignored and users will seek easier paths for processing their data. For this reason, the PSI has spent considerable effort developing and promoting the development of such software that implements the PSI standards.

Open-source software Application Programming Interfaces (APIs) in Java have been developed for most of the data standards. This is essential to promote adoption and to provide developers to include support for the standards in tools. These software libraries provide reading/writing functionality and other extra features. They are jmzML,^54^ jTraml,^55^ jmzIdentML,^56^ jmzQuantML,^57^ mzidLibrary,^32^ and jmzTab.^58^ Many open-source software tools implement the standards. Some of them were developed in Java making use of these libraries, but other programming languages have also been used such as Python or C.^59^

Perhaps the most difficult hurdle is the conversion of proprietary vendor binary formats to open standards. Initial attempts to reverse-engineer the vendor formats proved difficult but possible.^37^ However, as the vendors changed their formats to support new instrumentation, it became increasingly difficult to maintain the reverse-engineered parsing. Instead, the successful paradigm was to work with the vendors to produce a consistent API in the form of dynamic link libraries (DLLs) to access the data in the binary formats as implemented in ProteoWizard.^60^^,^^61^ This enabled a system of open-source libraries that could uniformly access data in the vendor binary formats, allowing direct access to data in the files, either for direct use by software or for conversion to an open standard format.^62^ Although direct access is seemingly preferable, in reality, the vendors have only provided Microsoft Windows-based (Microsoft, Redmond, Washington) DLLs, and thus direct access software must run on Windows or potentially Windows emulators. If the conversion strategy is used, then only the conversion must happen on the Windows-based computer, but all downstream processing may occur under other operating systems such as Linux or OS X.

In the context of MS proteomics databases, the ProteomeXchange consortium (http://www.proteomexchange.org)^63^ of proteomics resources has been recently established to develop standard submission procedures and dissemination pipelines of proteomics data in public databases, promoting data sharing practices in the field. At the moment of writing, it includes the resources PRIDE, PeptideAtlas (including PASSEL),^64^^,^^65^ and MassIVE (http://proteomics.ucsd.edu/ProteoSAFe/datasets.jsp). The consortium started as 1 output of the PSI group, and as such, one of the objectives of the consortium is to promote the use of PSI standards for storing the data.

In the field of molecular interaction databases, all major resources now make downloads available in the PSI-MI formats. These include primarily protein-protein databases such as IntAct,^9^ MINT,^9^ and DIP;^10^ drug-target databases such as ChEMBL^66^ and DrugBank;^67^ and pathway resources like Reactome.^68^ The IntAct database has also shown the format to handle more complex data types such as transcription factor-transcribed gene associations and the encyclopedic description of protein complexes. The use of shared data formats and CV terms has resulted in annotation/data gathering procedures becoming increasingly standardized across the different data resources, improving the user experience and making data integration much easier. Common standards have also enabled web services to be built across collaborating data resources, and a single query using the PSICQUIC query interface^69^ searches data in 31 different databases before presenting the user with a single collated response. The closer working relationship between many of the interaction databases resulted in the formation of the International Molecular Exchange (IMEx) Consortium^70^ to promote higher standards in data curation and to gather in the vast wealth of interaction information scattered across the scientific literature to be managed in a collaborative, nonredundant manner.

### Dissemination

While the creation of high-quality software tools has been a major factor in adoption of the PSI standards, other forms of dissemination have also played a key role. Although the activities of the PSI are largely led and implemented by academic researchers and members of their labs, vendor participation has been an important component. Involving the vendors in the design phases and the implementations of the standards encourages them to implement support for the standards in their own software, which leads to greater interoperability between commercial and open-source software as well as greater use of the standards in general.

Journal editor participation has been a key component of the dissemination efforts of the PSI. Not only have the editors been active participants in the development of minimum information guidelines, but they have also encouraged the formal publication of the standards and the tools that implement them in journal articles. In fact, there have been 10 or more journal articles published by the PSI each year over the past 5 years (for a comprehensive list see the PSI profile at Google Scholar at http://scholar.google.com/citations?hl=en&user=oNoChlcAAAAJ).

The PSI regularly hosts a workshop at each annual HUPO World Congress. These workshops typically consist of a few short presentations on the recent progress and ongoing efforts of the PSI followed by an opportunity for discussion and feedback with the audience. Other dissemination activities include hands-on training courses, in which the use of data standards is taught to mainly PhD students and postdocs, as part of broader courses on Proteomics Informatics or Network and Pathway analysis techniques.

The PSI has also engaged in efforts to spread its standards and methods for creating them to other disciplines. For example, the PSI has recently reached out to the metabolomics community. The mzML format is now being used in the metabolomics field after minor enhancements. The tab-delimited format mzTab supports metabolomics results in its first stable version. In addition, a group of stakeholders is currently working towards improving metabolomics data representation in mzTab (foreseen for the next version 1.1). The Coordination Of Standards In Metabolomics^71^ group is developing the nmrML format for storing the output of nuclear magnetic resonance (NMR) instruments following the methodology of the PSI. Similarly, the Molecular Interaction group has influenced the development of standards in the fields of protein affinity reagent production and of bioactive molecules such as drugs, herbicides, pesticides, and nutrachemicals.

All of the products of the PSI, including guidelines, format specifications, schemas, examples files, etc, may be examined and downloaded at the PSI website primarily. Some products are also hosted at central clearinghouses for similar products. For example, the PSI CVs are also distributed at BioPortal,^72^ the OBO Foundry,^73^ and the Ontology Lookup Service.^74^

## DISCUSSION

### Minimum Information Guidelines

The minimum information guidelines developed by the PSI (described above) have been useful but not adopted as widely as originally hoped. A few of the proteomics journals mention MIAPE as a recommendation, but none requires it. The journal *Molecular and Cellular Proteomics* (MCP) has developed its own set of guidelines.^33^ These guidelines are similar in many ways but also subtly different in aims. While the MIAPE guidelines are designed to describe what was done without imposing restrictions on minimal-quality metrics, the MCP guidelines extend further into providing minimal-quality guidelines and requirements for easily viewable spectra in cases where protein or post-translational modification detection evidence is not solid, in an effort to reduce the publication of spurious results. The MIAPE guidelines have been used as a guide for the development of software applications^31^ but is not universally applied. In some cases, it is viewed as being too demanding, but its low adoption mostly has been due to the lack of elegant software to make it easy for users to capture, manipulate, and edit metadata conforming to the MIAPE guidelines. If many more software applications would implement it in a way that is easy for users, adoption would increase significantly. When such software applications become widely available, the journals would likely feel more confident about making MIAPE compliance a requirement.

### Formats

The primary formats of the PSI have all been implemented in XML. The main reasons are that there are many tools in all languages to read, write, and validate XML, and that XML is text-based and thus human readable. This human readability has many benefits. It facilitates the development of the formats as the example data files can be easily viewed and discussed. It is easy for developers to resolve parsing problems by examining the files with a text viewer to determine what parts of the file are causing unexpected problems. In the case of minor errors or corruptions, a simple text editor can often be used to correct the problem and render the file readable once again. Finally, since the format is text-based and there are many open source XML parsers, these formats will remain readable in perpetuity. The primary drawback is that XML files are inefficient in terms of file size. However, disk space is relatively cheap compared with developer time and human effort; thus, this seems like a small cost. Further, these formats can be compressed with zlib, for example, although this renders some of the benefits described above somewhat tenuous. Other technologies similar to XML such as Resource Description Framework (RDF) were considered early on during the development of mzML, but these were dismissed in favor of XML primarily on account of unfamiliarity with RDF to the available developers, RDF's event greater verbosity, and RDF's apparent better suitability to metadata than raw data.

The landscape of MS proteomics is changing rapidly; therefore, it is necessary that the PSI formats are able to adapt to these changes. One option is to maintain a tightly controlled schema and update the schema frequently as new techniques, instruments, or workflows become available. However, there was great desire, especially from commercial companies, for the schemas to remain stable for a long time. The solution that was agreed upon was to create stable schemas that could be maintained for long periods of time but allow flexibility in the CV terms that could be used in the document. Thus, the format dictates where CV terms may be placed, but which CV terms are used can be flexible and can be adapted over time. As new techniques, instruments, and workflows become available, they can be described in the CV and then immediately used in documents. Some software that compiles a specific version of the CV into the code would need to be recompiled, but software that dynamically uses the latest CV may be able to make reasonable use of new terms without any changes. Validator programs for the PSI formats ensure that the CV terms are used in proper locations inside the data files.

In recent years, the first tab-delimited files have been developed. The motivation behind the development of formats like mzTab and MITAB was that tab-delimited formats had been previously successfully applied in other bioinformatics fields (for instance, the MAGE-TAB^75^ format in the microarray community), and it was perceived as a way to gain users (since some bioinformaticians prefer to implement tab-delimited file formats and are not so motivated to do it with XML-based formats) and to facilitate the reuse of proteomics data in other biological fields via easy export into MS Excel (Microsoft, Redmond, Washington) or R.

It has been commented that the sheer number of different formats produced by the PSI is confusing and that fewer formats would be less confusing. Current development has generally followed the paradigm of 1 format per data type. Since there are many different data types, many formats have been produced. However, since most of the XML formats follow a similar design methodology, there would be no major hurdles to combining the schemas to produce a format that could contain several data types. However, opinions vary on whether this would be beneficial or not, and there is no clear best path forward. However, since file sizes are already a problem in many cases, random access in XML files to pull out the needed information quickly is awkward, and all software would have to be adapted to read yet another format; creating a single format with many components for different data types seems impractical.

### Funding of activities

For the first 8 years of the operation of the PSI, there was essentially no stable funding for PSI development. All leaders and participants were contributing their time to the perceived laudable goals of the PSI. Sometimes these goals were well aligned with individual grants that paid the contributors. The workshops were funded by contributions from the vendors and other commercial companies that saw it beneficial to fund these workshops, both from the standpoint of having a “seat at the table” where the standards were developed and as a marketing benefit in general. In 2006, the European Union (EU)–funded Proteomics Data Collection Consortium began partially funding personnel to work on PSI standards. Starting in 2009, first the PSIMEx and then the ProteomeXchange FP7 EU coordination grants were funded, which explicitly included funding for personnel for software and standards development as well as funding for the workshops and other coordination activities. In 2013, as the ProteomeXchange grant was ending, the new UK Biotechnology and Biological Sciences Research Council (BBSRC)-funded “PROCESS” grant was obtained to continue funding some PSI activities. This was followed in 2014 by the “MIDAS” grant, also funded by the BBSRC, to support molecular interaction activities. The main conclusion is that while it has been possible to obtain funding for workshops from commercial companies with an interest in the formation of robust standards, funding from granting agencies for personnel and workshops has been crucial for the progress of the PSI.

### Interactions with other efforts

The interactions between the PSI and other consortia have provided great synergies and benefits to all parties. The PSI has been most closely working with the ProteomeXchange^63^ and IMEx consortia, since many of the most active PSI participants are also key personnel within 1 of these 2 consortia. The consortia are better able to accept and manage data because of the existence of standard formats, and the PSI benefits from increased participation and adoption of the standards it produces. Similarly, synergies between the PSI and the Proteome Project (HPP)^76^^,^^77^ have benefited both groups. The HPP has committed to make all data produced by participants publicly available via public repositories using PSI formats, and the PSI gains in visibility and adoption of its standards. Once the standards are used as part of 1 data submission or contribution to the HPP, they are more likely to be used in other unrelated projects as well. Several PSI formats have been adopted for use by metabolomics data pipelines and repositories. This has the following advantages: (1) The metabolomics community can benefit directly from lessons learned and extensive standardization work by the PSI. (2) Software can be reused in metabolomics contexts without major changes. (3) Data integration becomes easier when data formats are shared between 2 data types. (4) The PSI formats become more generally applicable and widely used. Such interactions between the PSI and other groups have been a general benefit to the whole field of biomedical informatics.

### Challenges for the future

Despite the many successes of the PSI, there remain many important challenges that need to be addressed. Most of the original goals of the PSI have been realized. We now have a set of stable approved standards for the major proteomics data types. What then remains? New needs, of course, will continue to emerge. For example, there is a growing demand for high-quality libraries of previously identified spectra, and there is no standard for this data type; thus, this is an obvious next step. However, moving beyond the creation of additional data standards to meet the needs of advancing technologies, the PSI faces additional challenges to maintain its past effectiveness. Despite the general success in achieving reasonable consensus and producing high-quality standards, adoption of the standards is not universal. The PSI should focus on fostering the development of better software that makes use of the standards easier. A few journals have developed their own data quality guidelines, and it would be beneficial for everyone if the PSI and existing journal guidelines could be reconciled. As discussed above, there does exist a perception by some that the PSI has created too many, varied formats, and the community would be better served by fewer formats that can hold more data types.

Several of our standards are applicable to other fields, and it would be useful to work with other communities beyond proteomics to achieve standards that can be applied to multiple disciplines. This has the effect of simplifying data analysis for all users. Finally, it will be a challenge to maintain healthy participation in the PSI. In earlier years, when the need for data standards was acutely visible, the PSI was easily able to recruit new programmers interested in developing important standards. But now, with many prominent standards in its portfolio, the need to join the PSI seems less compelling. Indeed, despite a repeated message that the PSI welcomes the participation of new members, there is a growing perception that those who have worked on these initial standards are the PSI, and so there seems less motivation for others to become a part of the initiative. Meeting these challenges will be required to expand the contributions of the PSI.

## CONCLUSION

The PSI has been very productive over the past dozen years and has helped accelerate the field of proteomics by greatly facilitating data sharing and data deposition in public data repositories. This has occurred through the coordinated development of minimum information guidelines, standard data formats, and, most importantly, software that implements these guidelines and formats. The success in proteomics is now attracting interest in other fields such as metabolomics, which will facilitate the integration of metabolomics data with proteomics data and applications to personalized medicine. Synergies with the MIBBI project^78^ are creating a bridge to entry points in medical informatics.

As technology advances over the next dozen years, the PSI will need to adapt current standards to remain relevant to these advances and forge new standards to enable data sharing and the interoperation of different software packages. Already, new workflows such as SWATH-MS^79^ and other DIA technologies are poised to revolutionize data collection, but analysis software for such workflows is still in its infancy and will benefit from standard formats so that different tools are interoperable. Further, coordination of standards with other omics technologies via similar standards organizations will greatly facilitate multiomic data integration.

## FUNDING

This work has been funded in part by EU FP7 grant ‘ProteomeXchange’ (grant No. 260558). EWD is also funded in part by NIH/NIGMS grant No. R01GM087221, the Center for Systems Biology/2P50GM075647, and the American Recovery and Reinvestment Act through the NHGRI grant No. RC2HG005805. ARJ and HH would like to acknowledge funding from the BBSRC (BB/K01997X/1). SO and HH would like to further acknowledge funding from the European Commission grant Affinomics (FP7-241481) and NHLBI Proteomics Center Award (HHSN268201000035C). ME is funded by PURE (http://www.pure.rub.de, Protein Unit for Research in Europe), a project of Nordrhein-Westfalen, a federal state of Germany. GM is funded by the Deutsche Gesetzliche Unfallversicherung project Deutsche Gesetzliche Unfallversicherung-Lunge (617.0 FP 339A). JAV is supported by the Wellcome Trust (grant No. WT101477MA) and the EU FP7 PRIME-XS (grant No. 262067).

## CONTRIBUTORS

All coauthors contributed to the writing of this article.

## COMPETING INTERESTS

None.
